# Assessing the clinical implications of low-density lipoprotein cholesterol equations using Nigerian data

**DOI:** 10.4102/ajlm.v14i1.2729

**Published:** 2025-09-19

**Authors:** Modupe A. Kuti, Jokotade O. Adeleye, Joshua O. Akinyemi, Olajumoke A. Ogundeji, Olusola O. Omoyele, Oluwadamilare A. Obe, Ademola S. Adewoyin, Oyetunji O. Soriyan

**Affiliations:** 1 Department of Chemical Pathology, Faculty of Basic Clinical Sciences, College of Medicine, University of Ibadan, Ibadan, Nigeria; 2 Synlab Nigeria Laboratories, Lagos, Nigeria; 3 Department of Medicine, Faculty of Clinical Sciences, College of Medicine, University of Ibadan, Ibadan, Nigeria; 4 Department of Epidemiology and Medical Statistics, Faculty of Public Health, College of Medicine, University of Ibadan, Ibadan, Nigeria; 5 Department of Chemical Pathology, University College Hospital, Ibadan, Nigeria; 6 Department of Chemical Pathology, Faculty of Basic Clinical Sciences, Lagos State University College of Medicine, Lagos, Nigeria; 7 Department of Medical Microbiology, Faculty of Basic Clinical Sciences, Lagos State University College of Medicine, Ikeja, Nigeria; 8 Department of Haematology, Faculty of Basic Clinical Sciences, College of Medicine, University of Lagos, Idi Araba, Nigeria; 9 Department of Chemical Pathology, Faculty of Basic Clinical Sciences, College of Medicine, University of Lagos, Idi Araba, Nigeria; 10 Department of Chemical Pathology, Lagos University Teaching Hospital, Idi Araba, Nigeria

**Keywords:** Calculated Low Density Lipoprotein Cholesterol, Friedewald formula, Martin-Hopkins equation, Sampson-NIH equation, Lipid profile, LDL cholesterol clinical categories

## Abstract

**Background:**

Newer equations, which are more accurate than the Friedewald formula (FF), have been published for the calculation of low-density lipoprotein (LDL) cholesterol. The impact of their adoption on decision-making has not been examined in Nigerian laboratories.

**Objective:**

This study examined the clinical implications of differences in estimating LDL cholesterol by the FF, Martin-Hopkins (MH), and Sampson-National Institutes of Health (NIH) equations.

**Methods:**

Between 01 January 2019 and 31 December 2023, lipid profile data, and the associated gender, were retrieved from the laboratory information system of Synlab Nigeria for persons aged 18–75 years. Differences in LDL cholesterol estimates from the three equations, and agreement with category assignments that determine clinical decisions, were examined.

**Results:**

Lipid profile data from 19 126 records were retrieved. This included data from 8234 (43.1%) women. The difference between FF estimates of LDL cholesterol and the other two equations was less than 10% for over 96% of the data. This difference increased with triglyceride levels. There was at least substantial agreement in the clinical category assignment of the equations, (ĸ > 0.715, *p* < 0.001). However, when triglycerides were > 1.69 mmol/L, the FF classification of < 1.81 mmol/L was classified as > 1.81 mmol/L in 43.3% and 25.1% of cases by MH and Sampson-NIH, respectively. For triglycerides > 4.51 mmol/L, there was constant bias, with MH higher than Sampson-NIH.

**Conclusion:**

Using the FF formula may significantly impact primary prevention of atherosclerotic cardiovascular disease. Switching to the MH or Sampson-NIH equation is advisable.

**What this study adds:**

This study provides a basis for Nigerian laboratories to switch from the Friedewald formula to one of the newer equations for the calculation of LDL cholesterol.

## Introduction

Low-density lipoprotein (LDL) cholesterol is a measure of the cholesterol mass transported within LDL and plays a prominent role in the pathogenesis of atherosclerotic cardiovascular disease. Although it can be measured directly in the laboratory, laboratories usually calculate LDL cholesterol using the Friedewald formula (FF) in persons whose plasma triglyceride concentrations are below 4.52 mmol/L.^1^ The FF obviates the need for the time-consuming, laborious and expensive preparative ultracentrifugation previously required for the determination of LDL cholesterol.^2^ Furthermore, the ease of using the formula, its cost effectiveness, convenience and relatively good accuracy in most clinical scenarios led to its global adoption and continued wide usage in most routine laboratories over the last 50 years.^3^ The availability of direct assays for LDL cholesterol in the late 1990s had minimal impact on this practice, as their introduction to laboratory operations meant an additional testing step and, consequently, increased costs. These direct assays also demonstrated susceptibility to significant interferences from hypertriglyceridaemia.^4,5^

There are, however, challenges with the use of the FF. Firstly, its inability to accurately estimate LDL cholesterol when the triglycerides are ≥ 4.52 mmol/L means that the laboratory needs to have an alternative means of determining and reporting LDL cholesterol in such scenarios. It has also been shown that the assumption in the formula of a fixed ratio of triglyceride levels to very low-density lipoprotein (VLDL) cholesterol of 5:1, when triglycerides are < 4.52 mmol/L, is physiologically inaccurate. According to the Lipid Research Clinics Prevalence Study, this ratio may actually vary between 5 and 9.^6^ A further implication of the above is that the FF may not be suitable when chylomicrons are present in the circulation. This may be particularly problematic with the increasing adoption of non-fasting specimens for initial lipid testing.^7,8^ The FF is also unsuitable for use in persons with dysbetalipoproteinaemia (type III hyperlipoproteinaemia).^2^

In response to these limitations, several alternative equations have been developed. Of these, the Martin-Hopkins (MH) equation^9^ and the Sampson-National Institutes of Health (NIH) equation^10^ are the most recent and pre-eminent. Unlike the FF, these two equations do not use a fixed factor for the triglycerides:VLDL cholesterol ratio. The original MH equation, published in 2013,^9^ determines the factor based on the ratio of triglyceride to non-high-density lipoprotein (HDL) cholesterol, and could be used with triglycerides levels up to 4.51 mmol/L. An updated version of the MH equation, which is able to report LDL cholesterol up to a triglycerides of 9.04 mmol/L, was subsequently released in 2021.^11^ The Sampson-NIH equation, released in 2020, also determines VLDL cholesterol from the triglycerides and non-HDL cholesterol, and has been reported to be accurate even with triglycerides up to 9.04 mmol/L.^10^ The reliability and accuracy of these equations is also contributed to by the fact that their derivation involved the use of much larger sample sizes and study populations that were more diverse with regard to health status and LDL cholesterol concentrations compared to those used for the FF.^1,9,10^

The majority of the laboratories in Nigeria continue to use the FF for the calculation of LDL cholesterol. For samples with a triglycerides value exceeding 4.51 mmol/L, either direct LDL cholesterol assays are employed, or no value is reported for LDL cholesterol and a comment is entered into the report. Adoption of either of these two formulae (MH or Sampson-NIH) would mean that the laboratory would be able to report LDL cholesterol for a larger percentage of its clients without additional cost. This study compared LDL cholesterol results obtained with the MH and Sampson-NIH equations against that of the FF. It examined any clinically significant differences in the results obtained with the three equations from lipid profile values from a Nigerian population. Furthermore, for samples with a triglycerides value exceeding 4.51 mmol/L, an assessment of the comparability of the results obtained with the MH and Sampson-NIH equations in this population was also performed.

## Methods

### Ethical considerations

This study was approved by the Lagos University Teaching Hospital, Health Research Ethics Committee on 29 November 2024, and was performed in accordance with the Declaration of Helsinki. This was a review of historic data which did not involve contact with any human participants. Patient consent was therefore not deemed required by the ethical board. All data were de-identified to maintain confidentiality and were stored on password-protected devices. Only de-identified data were used for analysis.

### Study design and source of data

This was an observational cross-sectional study. Data were retrieved from the laboratory information management system of Synlab Nigeria. All samples submitted to Synlab Nigeria for lipid studies between 01 January 2019 and 31 December 2023 were included in the analysis. Only data for persons aged between 18 and 75 were included in the final analysis. The data sets were filtered so that only the first (i.e. the oldest) lipid profile data per patient was included in the analysis.

Synlab Nigeria was selected for several reasons. It is the largest private medical laboratory in Nigeria with the widest national footprint. It maintained its ISO 15189 accreditation during the period of interest, and it receives samples collected from a diverse range of persons accessing care at primary, secondary and tertiary healthcare institutions. The Easymedical® Laboratory Information System, developed by Easy Medical SRL (Cluj-Napoca, Romania), is health-level 7 compatible and provides comprehensive information processing services for Synlab Nigeria Laboratories. The laboratory information system supports the electronic processing of all reportable patient testing performed by its laboratory including clinical, anatomic, and cytology test results. All completed test results are transmitted electronically from the performing machine to the laboratory information system for review and sharing with patients and doctors. Data on the laboratory information management system are stored both in a cloud-based server and terrestrially.

### Sample collection

Samples for lipid profile studies are collected within Synlab Nigeria into serum separator tubes. Samples from referring institutions may be either serum or ethylenediaminetetraacetic acid plasma. Individuals are normally advised to fast for at least 12 hours before presenting for sampling.

### Laboratory measurements and LDL cholesterol calculation

Total cholesterol, HDL cholesterol, and triglycerides were measured on the Beckmann AU680 clinical chemistry analyser (Beckman Coulter, Inc., Brea, California, United States). Low-density lipoprotein cholesterol is routinely calculated by FF for triglycerides < 4.52 mmol/L. No value is provided when triglycerides are ≥ 4.52 mmol/L.

The LDL cholesterol is calculated by the FF thus: LDL cholesterol = total cholesterol – HDL-cholesterol – (triglycerides/2.2) [mmol/L]. An Excel template for the calculation of LDL cholesterol by MH was graciously provided by Professor Seth Martin, while that for Sampson-NIH equation was calculated from an Excel template downloaded from https://nih.figshare.com/articles/code/Equation_Calculator_for_Low-Density_Lipoprotein_Cholesterol/11903274. The rendering of the Sampson-NIH equation is LDL cholesterol = total cholesterol/0.948 – HDL cholesterol/0.971 − (triglycerides/3.74 + [triglycerides*non-HDL cholesterol]/24.16 − triglycerides^2^/79.36) − 0.244 [mmol/L].

All samples with a triglycerides value > 9.04 mmol/L were excluded from the analysis.

### Statistical analysis

Data are presented as mean (standard deviation) or median (interquartile range), depending on normality of the data. Normality of data was assessed using the Kolmogorov-Smirnov Test. Differences in means were determined using the independent Student *t* test, and for medians using the Mann Whitney *U* test. Differences in the LDL cholesterol estimates of the equations was assessed using the nonparametric Wilcoxon Signed Rank Test. To assess the practical difference in interpreting cholesterol results, the Kappa coefficient was used to assess the degree of agreement in classifying individuals into LDL cholesterol categories. For lipid profile data beyond the 4.51 mmol/L limit of the FF, the agreements between the LDL cholesterol estimates by the MH and the Sampson-NIH equations were assessed by Spearman’s (rho) correlation coefficients and the Bland-Altman plot. All analyses were performed using the SPSS Statistics for Windows, Version 28.0 (IBM Corp., Armonk, New York, United States). Results were considered significant if *p* was 0.05 or less.

## Results

### Data characteristics

A total of 56 669 lipid profiles were received and analysed during the study period. After removing data for repeated visits, 19 126 remained for persons between the ages of 18 and 75 years (Table 1). The mean age of the women was 51.43 (s.d.: 12.99) years, and the mean age of men was 51.32 (s.d.: 12.56) years. Persons aged between 35 and 64 years provided 72.42% of the data. For total cholesterol, 10 205 (53.4%) of the values were less than 5.17 mmol/L, and for triglycerides, 17 924 (93.7%) of triglycerides values were less than 2.26 mmol/L. Regarding HDL cholesterol, 2561 (30.62%) women had values below 1.29 mmol/L, while 2328 (21.37%) men had values below 1.03 mmol/L. For lipid profiles with triglycerides < 4.52 mmol/L, the median (interquartile range) of all the LDL cholesterol estimates for the FF, MH, and Sampson-NIH equations were 3.10 (1.42) mmol/L for FF, 3.08 (1.40) mmol/L for MH, and 3.13 (1.45) mmol/L for Sampson-NIH, *p* < 0.001.

**TABLE 1:** Characteristics of lipid profile data obtained from Synlab Nigeria Laboratories between 01 January 2019 and 31 December 2023.

Participant characteristics	Female				Male				Total			
	*n*	%	Median	IQR	*n*	%	Median	IQR	*n*	%	Median	IQR
**Age groups (years)**	8234	43.05	-	-	10 892	56.95	-	-	19 126	100.00		
18–24	167	2.03	-	-	196	1.80	-	-	363	1.90	-	-
25–34	724	8.79	-	-	812	7.46	-	-	1536	8.03	-	-
35–44	1714	20.82	-	-	2419	22.21	-	-	4133	21.61	-	-
45–54	1990	24.17	-	-	2849	26.16	-	-	4839	25.30	-	-
55–64	2139	25.98	-	-	2741	25.17	-	-	4880	25.52	-	-
> 65	1500	18.22	-	-	1875	17.21	-	-	3375	17.65	-	-
**Cholesterol measurements**												
Total cholesterol (mmol/L)	-	-	5.12	1.63	-	-	4.83	1.63	-	-	4.94	1.63
Triglycerides (mmol/L)	-	-	0.89	0.54	-	-	1.02	0.71	-	-	0.96	0.63
HDL cholesterol (mmol/L)	-	-	1.45	0.46	-	-	1.24	0.39	-	-	1.32	0.44

s.d., standard deviation; IQR, interquartile range; HDL, high-density lipoprotein.

### Differences in LDL cholesterol estimates

**FIGURE 1:**
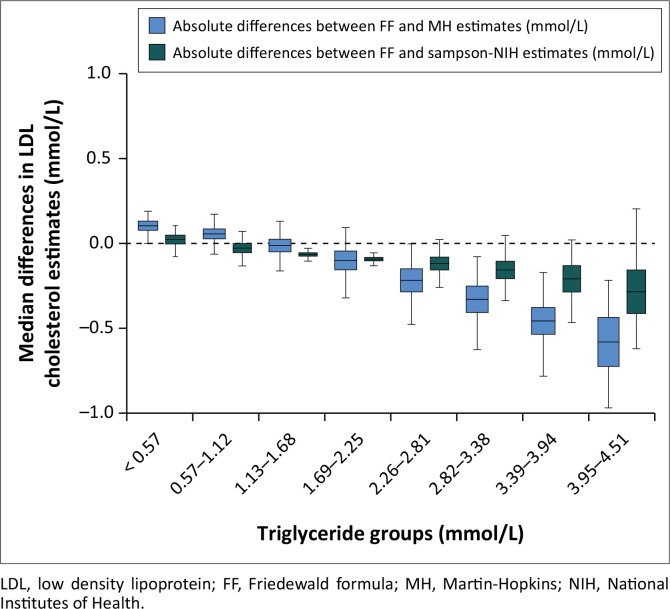
Median differences in LDL cholesterol estimates by triglyceride levels of lipid profile data obtained from Synlab Nigeria Laboratories between 01 January 2019 and 31 December 2023.

The percentage difference between LDL cholesterol estimates was less than 10% for the FF versus MH equations in 18 388 profiles (96.1%) (Table 2), and for the FF versus Sampson-NIH equations in 18 801 profiles (98.3%). The percentage difference exceeded 20% for FF versus MH in 258 profiles (1.3%), and for FF versus Sampson-NIH in 123 profiles (0.6%). The latter observations occurred when triglycerides were > 1.68 mmol/L. The median percentage difference between LDL cholesterol estimates of FF and MH, as well as between estimates of FF and Sampson-NIH, increased as triglycerides levels increased (Figure 1). The median percentage difference between the LDL cholesterol estimates of the equations exceeded 10% at triglycerides > 3.39 mmol/L for FF/MH, and at > 3.95 mmol/L for FF/Sampson-NIH.

**TABLE 2:** Percentage differences in LDL cholesterol estimates between the equations by triglyceride levels of lipid profile data obtained from Synlab Nigeria Laboratories between 01 January 2019 and 31 December 2023.

Triglycerides concentration (mmol/L)	*n*	FF – MH (percentage difference)			FF – Sampson-NIH (percentage difference)			MH – Sampson-NIH (percentage difference)		
		Median	IQR	*p*-value	Median	IQR	*p*-value	Median	IQR	*p*-value
< 0.57	1878	−4.15	2.39	< 0.001	−0.89	2.04	< 0.001	−3.59	2.33	< 0.001
0.57–1.12	10 190	−1.91	1.84	< 0.001	0.84	1.27	< 0.001	−2.73	1.80	< 0.001
1.13–1.68	4312	0.23	2.43	< 0.001	1.70	0.96	< 0.001	−1.46	1.89	< 0.001
1.69–2.25	1544	2.86	4.54	< 0.001	2.56	2.28	< 0.001	0.35	2.53	< 0.001
2.26–2.81	590	6.53	6.91	< 0.001	4.00	4.66	< 0.001	2.67	3.31	< 0.001
2.82–3.38	271	9.30	8.51	< 0.001	4.76	5.65	< 0.001	4.88	3.72	< 0.001
3.39–3.94	134	14.58	11.66	< 0.001	6.78	8.25	< 0.001	8.11	4.21	< 0.001
3.95–4.51	74	18.55	26.16	< 0.001	10.53	21.15	< 0.001	9.39	8.50	< 0.001
4.52–9.04	133	-	-	-	-	-	-	15.71	11.48	< 0.001

FF, Friedewald formula; MH, Martin-Hopkins; NIH, National Institutes of Health; IQR, interquartile range.

For triglycerides values between 1.69 mmol/L and 4.51 mmol/L, the Kappa coefficient was 0.715 (substantial agreement) for agreement between the LDL cholesterol estimates of FF and MH, and 0.81 (almost perfect agreement) for agreement between FF versus Sampson-NIH. Both coefficients were significant at *p* < 0.001. For LDL cholesterol classified as < 1.81 mmol/L by FF, 43.3% were classified as > 1.81 mmol/L by MH, and 25.1% were classified as > 1.81 mmol/L by Sampson-NIH (Table 3). Agreement with FF categorisation > 4.91 mmol/L was 100% for both MH and Sampson-NIH.

**TABLE 3:** Agreement of categorisations of the LDL cholesterol estimates at triglycerides concentration 1.69 – 4.51 mmol/L lipid profile data obtained from Synlab Nigeria Laboratories between 01 January 2019 and 31 December 2023.

FF Clinical treatment groups (mmol/L)	Clinical treatment groups (mmol/L)																							
	MH												Sampson-NIH											
	< 1.81		1.81–2.56		2.57–3.34		3.35–4.11		4.12–4.89		4.90		< 1.81		1.81–2.56		2.57–3.34		3.35–4.11		4.12–4.89		4.90	
	*n*	%	*n*	%	*n*	%	*n*	%	*n*	%	*n*	%	*n*	%	*n*	%	*n*	%	*n*	%	*n*	%	*n*	%
< 1.81	165	56.7	126	43.3	0	0.0	0	0.0	0	0.0	0	0.0	218	74.9	73	25.1	0	0.0	0	0.0	0	0.0	0	0.0
1.81–2.56	0	0.0	316	61.1	201	38.9	0	0.0	0	0.0	0	0.0	0	0.0	395	76.4	122	23.6	0	0.0	0	0.0	0	0.0
2.57–3.34	0	0.0	0	0.0	495	75.8	158	24.2	0	0.0	0	0.0	0	0.0	0	0.0	548	83.9	105	16.1	0	0.0	0	0.0
3.35–4.11	0	0.0	0	0.0	0	0.0	531	91.4	50	8.6	0	0.0	0	0.0	0	0.0	0	0.0	533	91.7	48	8.3	0	0.0
4.12–4.89	0	0.0	0	0.0	0	0.0	0	0.0	314	82.8	65	17.2	0	0.0	0	0.0	0	0.0	0	0.0	370	97.6	9	2.4
4.90	0	0.0	0	0.0	0	0.0	0	0.0	0	0.0	192	100.0	0	-	0	0.0	0	0.0	0	0.0	0	0.0	192	100.0

Note: Kappa coefficient (*p* value) was 0.715 (*p* < 0.001) for FF versus MH, and 0.81 (*p* < 0.001) for FF versus Sampson-NIH.

FF, Friedewald formula; MH, Martin-Hopkins; NIH, National Institutes of Health.

**FIGURE 2:**
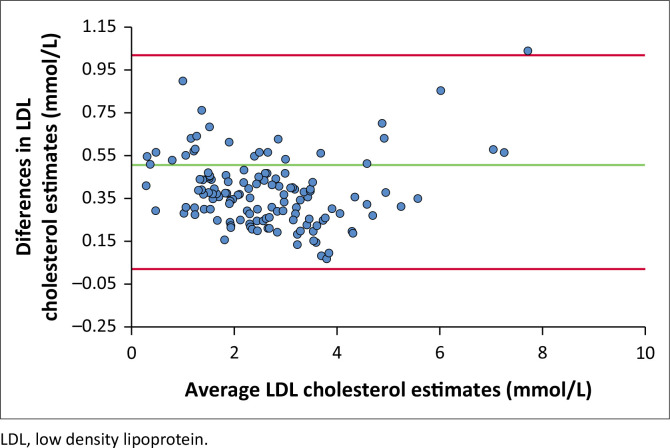
Bland-Altman difference plot of LDL cholesterol estimates by MH and Sampson-NIH at triglycerides > 4.51 mmol/L of lipid profile data obtained from Synlab Nigeria Laboratories between 01 January 2019 and 31 December 2023.

For the lipid profile data with triglycerides > 4.51 mmol/L, the correlation between the estimates for mean versus the difference of the MH and Sampson-NIH were strongly correlated; Spearman’s rho = 0.992 and *p* < 0.01. For the same data, the median (interquartile range) estimate of LDL cholesterol was 2.86 (1.62) mmol/L for MH and 2.41 (1.69) mmol/L for Sampson-NIH (data not shown). Figure 2 is a plot of the differences between MH and Sampson-NIH estimates against their means. It showed a constant bias with MH estimates higher than Sampson-NIH; however, almost all values were within the limits of agreement. The agreement of MH and Sampson-NIH LDL cholesterol categorisations had a Kappa score of 0.336, *p* < 0.001 (data not shown).

## Discussion

The findings in this study indicate that about 95.4% of the LDL cholesterol estimates by FF were within 0.26 mmol/L of those by the MH equation, and 99.4% for the Sampson-NIH equation. This represents a less than 15% difference for an LDL cholesterol concentration of 1.81 mmol/L. Furthermore, the recently updated set of quality requirements for acceptable performance in proficiency testing programmes released by the *Clinical Laboratory Improvements Act* has a target value + 20% for LDL cholesterol.^12^ Finally, estimates of the reference change value for LDL cholesterol, derived from biological variation data have been reported to be as high as 19%.^13^ The above suggests that, for the vast majority of LDL cholesterol estimates by the FF, the difference from the estimates by the newer equations may not have been clinically significant. This agrees with the observation of Wang et al. among a large US cohort.^14^ These would suggest that the Friedewald-calculated LDL cholesterol provides a reasonable estimation of LDL cholesterol and could guide treatment decisions for most patients, in addition to providing support for the recommendations of current guidelines on the continued use of this formula.

Our data revealed that, as triglycerides levels increased, the percentage difference between LDL cholesterol values calculated by FF and that calculated by MH increased. A similar pattern was observed between values calculated by FF and by Sampson NIH. This is probably because of the metabolism of VLDL, the main contributor to fasting plasma triglycerides, and the structure of the different formulae. As plasma triglycerides increase, there is a change in the relative concentrations of the two VLDL particle types. The proportion of large VLDL1 particles, which contain about 70% triglycerides, significantly outstrip that of the smaller VLDL2 particles, which consist of about 30% triglycerides. This difference in their relative concentrations widens with increasing triglycerides concentrations.^15^ Consequent to this, the FF, which has a fixed triglycerides:cholesterol ratio, will increasingly underestimate the concentration LDL cholesterol compared to the MH and Sampson-NIH, which adjust their estimate of VLDL cholesterol based on triglycerides concentrations. This triglycerides-influenced gradient in the difference between these equations has also been reported by others.^16,17^ Hypertriglyceridaemia of at least 1.68 mmol/L was present in over 1 in 10 of the profiles retrieved for this study.

In the context of primary prevention of atherosclerotic cardiovascular disease, hypertriglyceridaemia-related underestimation of LDL cholesterol could impact clinical decision-making. LDL cholesterol levels define the clinical category that is used to identify persons for whom statin therapy should be initiated, as well as those who are achieving the goal of therapy.^18^ From our data, about 50% of LDL cholesterol categorisation by FF as < 1.81 mmol/L, with triglycerides > 1.69 mmol/L, were classified in the > 1.81 mmol/L category by the MH equation. The implication of this is that persons either requiring the initiation or an intensification of therapy may not be offered the same. These findings are very similar to several other reports and indicate a major limitation to the continued and unqualified use of the FF for primary prevention of atherosclerotic cardiovascular disease.^19,20,21^ Recognising this limitation of the FF, guidelines on the management of blood cholesterol for the primary prevention of atherosclerotic cardiovascular disease recommend the measurement of direct LDL cholesterol or the use of another equation, such as the MH or NIH, to improve accuracy of risk assessment when LDL cholesterol is less than 1.81 mmol/L.^1,22^ Although present to a lesser degree, significant and clinically consequential underestimation of LDL cholesterol was also observed at higher LDL cholesterol concentrations and with the NIH equation.

For the clinical laboratory seeking to improve its support for atherosclerotic cardiovascular disease primary prevention efforts and to replace the FF formula, there are items to consider. Firstly, the laboratory must decide which of the several available formulae is to be adopted. A key consideration for this decision should be scientific evidence of superior analytical accuracy in determining the concentration of LDL cholesterol as compared to the gold standard method of beta quantification. With regard to the two pre-eminent equations used in this study, there are claims and counterclaims of superior accuracy. Concerns have been raised over accuracy of the variant of the gold standard technique used, the population used for the development of the formulae, as well as the basis and reliability of the factor used in estimating the cholesterol content of VLDL.^23,24^ For primary prevention efforts, what may be of more importance is the classification of individuals into the appropriate LDL cholesterol treatment category, especially in the above discussed context when there is hypertriglyceridaemia. From our data, although the level of agreement between the two equations with each other in this scenario was better than with the Friedewald equation, there were still significant differences, especially when LDL cholesterol was < 1.81 mmol/L. Very similar to our findings, Sajja et al.,^25^ following their review of the electronic health record data from patients with atherosclerotic cardiovascular disease, reported clinically significant discordance in a pairwise comparison of the Sampson-NIH equation and the MH equation of about 23% at LDL cholesterol values of ≥ 1.81 mmol/L. At triglycerides > 4.52 mmol/L, our data indicate only fair agreement between the two equations. These suggest that the LDL cholesterol estimates from these two equations are not interchangeable. Based on these, it has been suggested that laboratories should indicate on their report which equation they are using to calculate their LDL cholesterol estimates or, if feasible, report the estimates of multiple equations.^26^

Another major consideration for the laboratory seeking to replace the FF is the ease of implementing the new formula. The FF is a simple arithmetic formula that requires little expertise to incorporate into a laboratory information system software. The Sampson-NIH equation is a more mathematical bivariate quadratic equation developed by multiple least squares regression. The actual equation may require marginally higher expertise than the FF, but should still be easily implementable.^10^ The MH equation is more complex than the above two. It is similar to the FF, but uses an adjustable factor to divide the triglycerides instead of the fixed 5. It is rendered as LDL cholesterol = total cholesterol – HDL cholesterol – (triglycerides/adjustable factor). The adjustable factor is sourced from a 240-cell stratification of triglycerides and non-HDL cholesterol. Implementation of this formula would require some coding.^9^ In essence, implementation of the Sampson-NIH equation might be easier for the majority of laboratories.

### Limitations

The effect use of historic data which may have been sourced from a population that is diverse in clinical circumstances may be unpredictable. The size of the data employed for this study may have dampened the effect of this limitation.

### Conclusion

The FF is inadequate to support current efforts at primary prevention of atherosclerotic cardiovascular disease and should no longer be recommended by guidelines. Laboratories must switch to more modern equations to provide more reliable LDL cholesterol estimates. Depending on the capacity of the laboratory, the implementation of the Sampson-NIH equation might be easiest.
